# Crystal Structure of PAV1-137: A Protein from the Virus PAV1 That Infects *Pyrococcus abyssi*

**DOI:** 10.1155/2013/568053

**Published:** 2013-03-04

**Authors:** N. Leulliot, S. Quevillon-Cheruel, M. Graille, C. Geslin, D. Flament, M. Le Romancer, H. van Tilbeurgh

**Affiliations:** ^1^Institut de Biochimie et de Biophysique Moléculaire et Cellulaire, CNRS-UMR 8619, IFR115, Université Paris-Sud, Bâtiment 430, 91405 Orsay, France; ^2^Laboratoire de Cristallographie et RMN Biologiques-CNRS UMR-8015, Université Paris Descartes, Faculté des Sciences Pharmaceutiques et Biologiques, 4, av de l'Observatoire, 75270 Paris CEDEX 06, France; ^3^Laboratoire de Biochimie (BIOC), CNRS UMR 7654, Ecole Polytechnique, Route de Saclay, 91128 Palaiseau, France; ^4^Université de Brest, CNRS, IFREMER, UMR 6197, Laboratoire de Microbiologie des Environnements Extrêmes, OSU-IUEM, Technopôle Brest-Iroise, Avenue Dumont D'Urville, 29280 Plouzané, France

## Abstract

*Pyrococcus abyssi* virus 1 (PAV1) was the first virus particle infecting a hyperthermophilic Euryarchaeota (*Pyrococcus abyssi* strain GE23) that has been isolated and characterized. It is lemon shaped and is decorated with a short fibered tail. PAV1 morphologically resembles the fusiform members of the family Fuselloviridae or the genus *Salterprovirus*. The 18 kb dsDNA genome of PAV1 contains 25 predicted genes, most of them of unknown function. To help assigning functions to these proteins, we have initiated structural studies of the PAV1 proteome. We determined the crystal structure of a putative protein of 137 residues (PAV1-137) at a resolution of 2.2 Å. The protein forms dimers both in solution and in the crystal. The fold of PAV1-137 is a four-**α**-helical bundle analogous to those found in some eukaryotic adhesion proteins such as focal adhesion kinase, suggesting that PAV1-137 is involved in protein-protein interactions.

## 1. Introduction

The archaea domain is organized into two major phyla, the Crenarchaeota and the Euryarchaeota. The first phylum contains mainly the extremely thermophilic Sulfolobales, Desulfurococcales, and Thermoproteales. The vast majority of hyperthermophilic viruses were isolated from the Crenarchaeota infecting in particular the genera *Sulfolobus*, *Thermoproteus*, *Acidianus, Pyrobaculum, Stygiolobus, *and* Aeropyrum* [1, 2]. Their shapes are characterized by unusual morphologies very different from bacterialviruses and eukaryotic viruses. Genomic sequences were determined for some of these archaeal viruses and revealed a very high portion of ORFan genes [3]. Due to their exceptional morphological and genomic properties, they were assigned to eight novel viral families [1].

 The Euryarchaeota phylum includes extreme halophiles, methanogens, and hyperthermophilic sulfur reducers (*Thermococcales*). Most of the viruses infecting this phylum are isolated from mesophilic hosts and are tailed viruses, whereas pleomorphic types are relatively rare [4]. The knowledge about archaeal viruses is still very limited and this is even more poignant for viruses that infect hyperthermophilic Euryarchaeota [5, 6]. To date, PAV1 and TPV1 (*Thermococcus prieurii* virus 1) are the only viruses isolated from cultivated marine hyperthermophilic euryarchaea. These spindle-shaped viruses are morphologically similar to the haloviruses of the genus* Salterprovirus* [7, 8] that infect extreme halophiles, and to crenarchaeal viruses assigned to the fusiform family *Fuselloviridae* [9], but they do not share any genomic properties. PAV1, isolated from *Pyrococcus abyssi*, was the first virus isolated and described in *Thermococcales *[10]. PAV1 virions display a lemon-shaped morphology (120 nm long and 80 nm wide) with a short tail (15 nm) terminated by fibers. Very recently a novel fusiform virus, TPV1, was isolated and characterized from the hyperthermophilic euryarchaeal genus *Thermococcus* [11].

PAV1 and TPV1 are released during all phases of host growth without causing host lysis. A simple procedure to spot viruses on cellular lawns and directly observe their impact has been specially designed. This allows determination of the host range and infectivity of viruses isolated from anaerobic hyper/thermophile sulfur-reducing microorganisms. We used this approach to prove the infectivity of PAV1 and to confirm the host range of TPV1, both of them being genus specific [12]. 

 The genome of PAV1 is composed of a double stranded circular DNA. It was shown that the free viral genome exists as a multicopy plasmid in the host strain, but no integrated prophage could be detected. The complete genome of PAV1 contains 18,098 bp [13]. A number of 25 ORFs (open reading frames) encoding at least 50 amino acids were identified and almost all are located on the same strand. The shape of the viruses is perturbed upon treatment with organic solvents or detergents, suggesting that their envelopes contain lipids. This observation is supported by the fact that half of the PAV1 genome has predicted transmembrane helices. Sixty-five percent of the predicted proteins have no homologues in the sequence databases. Functions could only be vaguely suggested for three proteins. A 59 amino acid protein (PAV1-59) shares similarities to the CopG transcriptional regulators. Two other ORFs (PAV1-676 and PAV1-678) that are the only ORFs shared between the hyperthermophilic euryarchaeal viruses PAV1 and TPV1 contain one or two copies of the laminin G-like jelly roll fold and may hence be involved in adhesion to the host. Polycistronic mRNA analysis showed that all predicted genes are transcribed in six mRNAs.

 In contrast with other lemon-shaped viruses isolated either from hypersaline waters (*Salterprovirus*) or from extreme geothermal terrestrial environments (*Fuselloviridae*), PAV1 was isolated from a remote deep-sea hydrothermal vent. So, the uniqueness of the PAV1 genome compared to those of other archaeal viruses may be a consequence of its evolutionary history [14]. Since no function could be proposed for most of the predicted ORFs, we set out to analyze the structures of the proteins encoded by the PAV1 genome. The assignment of function to archaeal proteins suffers from the absence of genetic data and sequence analogs in better-characterized organisms [15–18]. 3D structure is better conserved than sequence and may reveal similarities that remain undiscovered by sequence analysis [19]. Therefore, structure determination offers a valuable alternative for investigating protein function. Indeed, the determination of the crystal structure of the AvtR protein from a hyperthermophilic archaeal lipothrixvirusallowed us to establish a role for this protein in the transcriptional regulation of viral genes [20]. We want *in fine* to find out if PAV1 protein structures are related to those of other archaeal virus proteins. We present here the X-ray crystal structure of a putative ORFan protein PAV1-137, to our knowledge the first for a euryarchaeal viral protein. 

## 2. Results and Discussion

We purified the C-terminal His-tagged protein from a genetic construct deleted for the 14 first residues because sequence analysis predicted an unstructured conformation for this N-terminal region [21]. MALDI-TOF mass spectrometry analysis of the purified recombinant protein shows that the N-terminal methionine was cleaved off during the production in *E. coli*. Gel filtration analysis suggests that the protein forms dimers in solution (not shown). Crystals were obtained in 35% PEG400, 0.5 M NH_4_Cl, 0.1 M Na citrate at pH 4, at a concentration of 15 mg/mL for the protein. Details of data collection and refinement are found in Table 1. All residues of the construct, except for the affinity tag, are visible in the electron density. Two copies of PAV1-137 are present in the asymmetric unit and their structures are almost identical (root mean square deviation = 0.5 Å). 

PAV1-137 contains four amphipathic helices that are organized as a helical bundle. The three N-terminal helices form a parallel up and down configuration while the C-terminal helix is shorter and packs with an angle of about 45° against helices 1 and 3. The core of the 3 helices is very hydrophobic consisting mainly of branched aliphatic amino acids. The connections between helices 1, 2, and 3 are short. The connection between helices 3 and 4 is a longer stretch, resulting in a less tight packing of helix 4 compared to the other helices. 

**Figure 1:**
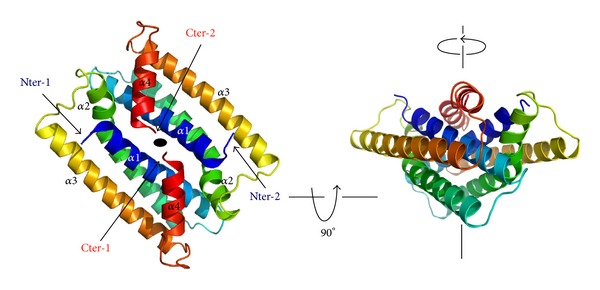
Two perpendicular views of the X-ray structure of the PAV1-137 dimer. The two subunits are represented in rainbow colouring going from blue (N-terminal) to red (C-terminal). The N and C terminus and the helices of the two subunits are labelled. The two-fold axis is indicated.

The A and B monomers in the asymmetrical unit form a two-fold symmetrical dimer and the symmetry axis runs perpendicular to the direction of the long helices (Figure 1). The helices of both monomers associate to form an antiparallel super helical bundle. The dimer interface involves helices 1, 2, and 4. The accessible surface of each subunit buried by dimer formation is substantial. The accessible surface area for the monomer is 7300 Å^2^. Dimerisation buries 1278 Å^2^ per monomer corresponding to 17% of the solvent accessible surface area. The majority of interactions between the monomers are conferred by helix 4 that lies against the extremities of helices 1 and 2. The interface is more hydrophilic than the core of the helical bundle and is stabilized by 9 hydrogen bonds, mainly between side chain and side or main chain atoms.

Orthologs of PAV1-137 were recently reported in genomic sequences of new thermococcus plasmids [14]. The gene coding for the ortholog of PAV1-137 is found in tandem with an ortholog of gene PAV1-375 in three of these plasmids. Surprisingly, it also formed a three-gene cluster, which is conserved within the provirus A3 VLP of the euryarchaeal methanogens *Methanococcus voltae* A3 [22]. PAV1-375 likely encodes for a P-loop ATPase, but its association with PAV1-137 remains unclear. PAV1-137 has presently no structural analogues in the Protein Data Bank that could help defining its function. The lack of sequence analogues at the start of this study suggested that PAV1-137 might adopt a new fold. Helical bundles are extremely common in protein structures. Therefore we found substantial structural similarities with proteins sharing helical bundle architecture. Examination of the function of these proteins clearly shows that the structural similarity does not indicate functional relationships. For example, significant overlaps are found between PAV1-137 and fragments of Talin and Focal adhesion kinase, both from eukaryotic origin (*Z*-score 5 and root mean square difference of 3.2 Å for 100 aligned residues) [23, 24]. Helices 1, 2, and 3 of PAV1-137 superpose well onto helices 2, 3, and 4 of the eukaryotic helical bundles. No equivalent is present in these eukaryotic analogues for the fourth helix found in PAV1-137. The C-terminal helix adopts a different orientation and has no equivalent in Talin or Focal adhesion kinase. 

Since PAV1-137 does not seem to carry any active site, its biological function is probably connected with protein-protein or protein-nucleic acid interactions. Helical bundle proteins are frequently involved in this type of interactions, exemplified by Talin. Virtually nothing is known about the life cycle of PAV1. Its genome sequence was a first step towards a better understanding of this new type of viruses which may have evolved from a recombination event between different mobile genetic elements harbored by both hyperthermophilic and methanogenic euryarchaeota. We report here on the first results of the investigation on structure and function of proteins encoded by this virus.

## 3. Materials and Methods

### 3.1. Protein Production and Purification

The PAV1-137 ORF lacking the region encoding for the 14 N-terminal residues was amplified by PCR using genomic DNA of PAV1 virus as a template. An additional sequence coding for a 6-histidine tag was introduced at the 3′ end of the ORF during amplification. The PCR product was then cloned into pET28 vector. Expression was done at 37°C using the *E. coli *BL21 (Gold)DE3 strain. The His-tagged protein was purified on a Ni-NTA column (Qiagen Inc.) followed by gel filtration using a buffer composed of 20 mM Tris-HCl pH 7.5, 200 mM NaCl, and 10 mM b-mercaptoethanol. Selenomethionine-substituted PAV1-137 was produced and purified as the native protein. The peak fractions were concentrated to 15 mg/mL and used for crystallization.

### 3.2. Crystallization and Data Collection

Crystallization was performed using a Cartesian crystallization robot in 200 × 200 *μ*l sitting drops (volume for protein and liquid mother) and reproduced manually in 1 × 1 *μ*L drops. Crystals were obtained from the following crystallization conditions: 35% PEG400, 0.5 M NH_4_Cl, 0.1 M Na citrate at pH 4 at 18°C. Crystals were transferred in the mother liquor containing 30% glycerol prior to flash freezing in liquid nitrogen. X-ray diffraction data of SeMet substituted protein crystals were collected on the ID14-4 ESRF beamline and were processed using MOSFLM and SCALA [25]. The crystals belong to the P6_1_22 space group with two molecules per asymmetric unit. The cell parameters and data collection statistics are reported in Table 1.

### 3.3. Structure Solution and Refinement

The structure was solved at a resolution of 2.2 Å by single anomalous diffraction (SAD) using data collected at the Selenium peak wavelength. The Hyss module of Phenix program [26] was used to find the Selenium sites using the entire resolution range. The sites were refined with SHARP [27], and solvent flattening was performed with DM. Arp/Warp [28] built 95% of the visible residues. The model was fully refined and completed from the native data using Buster and the graphics programme O [29, 30]. Refinement was carried out using noncrystallographic symmetry restraints and one TLS group per chain (statistics are shown in Table 1) with the program Buster. All the residues fall in favourable regions of the Ramachandran plot. 

For homology search, we did a Psi-BLAST analysis using standard procedures as provided by the NIH blastserver (http://blast.ncbi.nlm.nih.gov.gate1.inist.fr/Blast.cgi).

## Figures and Tables

**Table 1:** Data collection and refinement statistics.

Data collection	
Space group	P6_1_22
Cell dimensions *a*, *b*, *c* (Å)	73.55, 73.55, 191.55
Wavelength (Å)	0.9793
Resolution (Å)	20.0–2.20
Outer resolution shell (Å)	2.32–2.20
Number of observed reflections/unique	94031/15911
Completeness (%) (outer shell)	97.4 (85.3)
Multiplicity (outer shell)	5.9 (2.8)
*I*/*σ*(*I*) (outer shell)	19.0 (2.4)
*R*_merge_ (%)^1^ (outer shell)	5.8 (45.9)
Refinement	
Resolution (Å)	20.0–2.20
Reflections (working/test)	15873/800
*R*/*R*_free_ (%)^2^	21.5/24.7
RMSD	
Bond lengths (Å)	0.010
Bond angles (°)	1.15
B-factors (Å^2^)	
Protein	63
Ramachandran statistics (%)	
Most favored	98.71
Allowed	1.29

^1^ *R*_merge_ = ∑_*h*_∑_*i*_|*I*_*hi*_ − 〈*I*_*h*_〉|/∑_*h*_∑_*i*_ *I*_*hi*_, were *I*_*hi*_ is the *i*th observation of the reflection *h*, while 〈*I*_*h*_〉 is the mean intensity of reflection *h*.

^2^ *R* = ∑||*F*_*o*_| − |*F*_*c*_||/|*F*_*o*_|. *R*_free_: was calculated with a set of randomly selected reflections (5%).
